# Surface Plasmon Resonance Biosensors: A Review of Molecular Imaging with High Spatial Resolution

**DOI:** 10.3390/bios14020084

**Published:** 2024-02-02

**Authors:** Jiying Xu, Pengfei Zhang, Yi Chen

**Affiliations:** 1 National & Local Joint Engineering Research Center for Mineral Salt Deep Utilization, Faculty of Chemical Engineering, Huaiyin Institute of Technology, Huaian 223003, China; 2 Key Laboratory of Analytical Chemistry for Living Biosystems, Institute of Chemistry, Chinese Academy of Sciences, Beijing 100190, China; 3 University of Chinese Academy of Sciences, Beijing 100049, China

**Keywords:** surface plasmon resonance, surface plasmon resonance imaging, surface plasmon resonance microscopy, surface plasmonic scattering microscopy, molecular imaging, high spatial resolution

## Abstract

Surface plasmon resonance (SPR) is a powerful tool for determining molecular interactions quantitatively. SPR imaging (SPRi) further improves the throughput of SPR technology and provides the spatially resolved capability for observing the molecular interaction dynamics in detail. SPRi is becoming more and more popular in biological and chemical sensing and imaging. However, SPRi suffers from low spatial resolution due to the imperfect optical components and delocalized features of propagating surface plasmonic waves along the surface. Diverse kinds of approaches have been developed to improve the spatial resolution of SPRi, which have enormously impelled the development of the methodology and further extended its possible applications. In this minireview, we introduce the mechanisms for building a high-spatial-resolution SPRi system and present its experimental schemes from prism-coupled SPRi and SPR microscopy (SPRM) to surface plasmonic scattering microscopy (SPSM); summarize its exciting applications, including molecular interaction analysis, molecular imaging and profiling, tracking of single entities, and analysis of single cells; and discuss its challenges in recent decade as well as the promising future.

## 1. Introduction

Quantifying molecular interactions is critical for screening drugs, discovering disease biomarkers, and understanding biological processes at the molecular scale [1,2,3,4,5,6]. Surface plasmon resonance (SPR) utilizes surface plasmon polarizations and their waves propagating along noble metal film surfaces. Owing to the fact that surface plasmon waves can notably enhance light–matter interactions and be immune to the impurities away from the sensor surface by significantly reducing the illumination volume, the combination of SPR technology and biologically modified sensor surfaces enables label-free and highly sensitive detection of molecules in their natural forms, thus allowing quantitative studies of their interaction kinetics without the interference of the labels [7,8]. After decades of development, SPR technology has become a powerful tool for studying biomolecular interactions and is popular in diverse fields, including, but not limited to, biochemical research, clinical diagnosis, and the pharmaceutical industry [9,10,11,12,13,14,15,16,17,18,19,20,21,22,23,24,25,26,27].

To improve detection throughput, SPR imaging (SPRi) has been developed since the late 1980s [28,29]. SPRi can be built with either a prism or a microscopic objective. The former is usually based on the classical Kretschmann configuration, where the incident light obliquely illuminates the sensor surface via a glass prism with a total internal reflection configuration to excite the SPR on the noble metal surface and one area detector, such as a camera, is used to receive the reflected light to create the SPR images [30,31,32,33,34,35,36,37]. Due to the physical constraints resulting from the prism, it is impossible to use a high numerical aperture objective in the detection path to create SPR images. Furthermore, optical refractions on the prism–air interface introduce additional distortions in the SPR images. Consequently, prism-coupled SPRi sensors can usually only provide a spatial resolution larger than 10 μm [38,39,40,41]. To improve the spatial resolution, the latter was developed using an oil-immersed objective with a high numerical aperture based on the Kretschmann configuration, which is also referred to as SPR microscopy (SPRM) [42]. The SPRM excites surface plasmonic waves by directing light at an appropriate angle via an oil-immersion objective onto a gold-coated glass slide placed on the objective. Then, light reflected from the gold surface is collected to form an SPR image. SPRM image contrast is determined by the interference between the planar plasmonic wave and the plasmonic wave spherically scattered by the samples. Owing to the high numerical aperture of the objective, SPRM can reach a diffraction-limited spatial resolution of ~300 nm perpendicular to the propagation direction of surface plasmon waves, which has been widely used in the imaging of single nanoparticles [43,44,45,46] and single cells [23], subcellular organelles [47], virions [48,49], nanoparticles [50,51], nanobubbles and exosomes [52]. In addition, SPRM can also utilize the exponentially decaying properties of the electric fields of the surface plasmon waves to track the sample movements at the axial direction for quantitatively determining the interactions of nanoparticles or biological entities with the surface, thus providing a powerful tool to understand particle absorption mechanisms [53,54,55,56,57,58,59,60,61,62,63,64,65,66,67,68,69,70]. In addition, owing to the fact that the resonance condition of SPR depends on the refractive index near the sensor surface, SPRM can also be combined with electrochemical workstations for studying the spatial distributions of electrochemical reaction kinetics [71,72,73]. However, due to the finite decaying length of the surface plasmonic wave along the propagation direction (longitudinal), the SPRM images are accompanied by a parabolic tail that is many microns long, resulting in lower spatial resolution in the longitudinal direction [46,51,74,75,76,77,78,79,80,81,82,83,84,85].

In order to remove the parabolic tails for higher imaging resolution (e.g., down to sub-micrometers in all directions), an interference plasmonic imaging approach was first developed, where the fast Fourier transform algorithm was employed to process the original SPRM images, and filtering of the frequency domains was utilized to remove the parabolic tails [43,52,75]. However, this approach essentially relies on post-processing and cannot obtain high-spatial-resolution images directly, and it is also questionable in terms of spatially resolving naturally densely distributed samples, such as cell adhesion sites. To directly obtain SPRM images with high spatial resolution, the multidirectional or azimuthal rotation illumination approach was developed, where azimuthal averaging was used to neutralize the parabolic tails and mechanical scanning was used to create the images, but this method is accompanied by the tradeoff of temporal resolution and not suitable for analyzing molecular interactions and other dynamic processes [86,87].

A novel approach, surface plasmonic scattering microscopy (SPSM), was recently developed to directly achieve SPR images with high spatial resolution [88,89,90,91,92]. SPSM can be built on both conventional prism and objective coupled SPRi systems by adding one dry objective on the top of the sensor chips and directly collecting the surface plasmon waves scattered by the analytes so that they are free of interference from propagating surface plasmon waves with long decaying lengths to acquire SPR images without parabolic tails. Owing to this, SPSM can achieve diffraction-limited spatial resolutions at any lateral direction in real time. Furthermore, SPSM does not collect strong reflections that unavoidably contain non-resonant components, making it possible to provide high-contrast images for clear observations of the sample details. Additionally, SPSM permits incident light intensity up to kilowatt per centimeter, thus providing enough signal-to-noise ratio to image single proteins in a label-free manner.

Spatial resolution is vitally important for molecular imaging. While some reviews have been published targeting working principles, setups, and applications [55,93,94,95,96,97,98], they lack a focus on the increment of spatial resolution. In this minireview, we will start with an overview of the principles and instrumentation of an imaging methodology based on SPR. We then discuss their latest applications to biomolecular interactions. Finally, we address the current challenges in conducting SPR-based high-spatial-resolution imaging and discuss the perspectives on future developments.

## 2. Principle and Instrumentation

*Prism-coupled SPRi*. Prism-coupled SPRi is constructed by placing gold-coated cover glasses on a prism coupler based on the Kretschmann configuration [99]. A p-polarized light was directed onto the gold film surface with an appropriate angle to excite the SPR, creating surface plasmon waves with an electric field enhancement of 20–30 times within ~100 nm near the sensor surface. Consequently, SPR results in light absorption and the adsorption of molecules onto the gold film surface can result in effective refractive index changes within the electric field of surface plasmon waves, thus leading to variations in SPR conditions, which can be observed by monitoring the reflection light intensity. In a typical SPRi system, a collimated light beam is employed as the excitation light at a fixed angle, and the spatial distributions of samples can be detected by using a CMOS or CCD camera by tracking the variations in reflection light intensities [7]. Alternatively, one can also employ mechanical scanning techniques to construct a SPRi system, where angular interrogation or spectral interrogation can be used to achieve a wide linear dynamic range with tradeoffs in temporal resolution [100,101]. In addition, the adoption of a multiwavelength excitation source and color CCD produces color SPRi for more visualized analysis [102].

**Figure 1:**
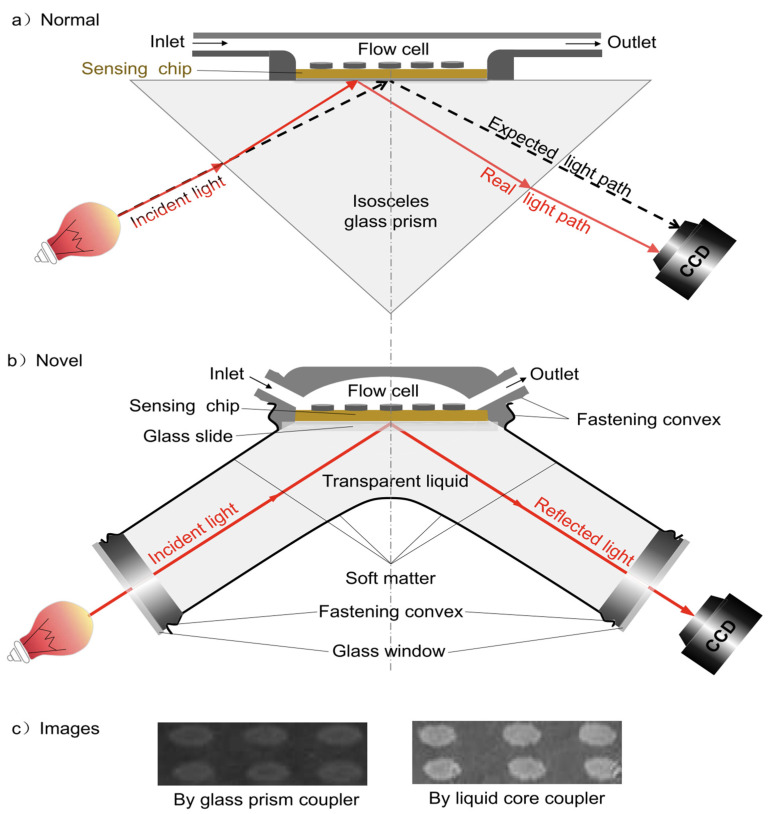
Schematic structure of SPRi based on a glass prism (**a**) and liquid core coupler (**b**), and direct SPRi images of BSA spots at spotting concentration of 1 mg/mL without further image processing (**c**). Reprinted from Refs. [104,107] with permission. Copyright Elsevier 2017.

One common issue for the prism-coupled SPRi is the image distortion caused by the refraction occurring on the prism surfaces along the propagation direction, which can be partially corrected by replacing the solid glass prism with a transparent liquid in a sealed soft cavity to ensure that the excitation light is always perpendicular to the incident window and the reflected light is always perpendicular to the exit window in order to reduce the interference of multiple interface reflection, therefore improving both image contrast and spatial resolution (Figure 1). Additionally, another advantage of liquid core coupling is adjustably enhanced SPR sensitivity with universal applicability [103,104,105,106]. Another issue of the prism-based SPRi is that the high numerical aperture objective can hardly be used due to the mechanical constraint, thus making it unable to achieve spatial resolution at the sub-micrometer level.

*SPRM.* A typical SPRM can be constructed in an inverted optical microscope [42]. A p-polarized monochromatic light beam is focused onto the back-focal plane of a high numerical aperture oil-immersion objective to illuminate gold-coated cover glasses at an appropriate angle to excite the SPR. Then, the reflected light can be collected by the same objective and imaged using a CCD or CMOS camera. Excellently, SPRM can provide sub-micrometer spatial resolution in the direction perpendicular to the propagating direction of surface plasmon waves. However, the surface plasmon waves scattered by the analyte can interfere with the propagating surface plasmon waves and then form parabolic tails together with the analytes. The imaging spatial resolution along the surface is largely reduced. Two typical approaches have since been employed to remove the parabolic tails. One is image reconstruction using the fast Fourier transform algorithm [43,75], which utilizes the special features of the parabolic tails in the frequency domain to remove them. It requires post-processing and cannot directly achieve high-spatial-resolution images. An alternative way is the multidirectional or azimuthal rotation illumination approach [86,87], which employs radially polarized light to excite SPR and to notably suppress the intensities of the parabolic tails by offsetting the interference among the surface plasmon waves in different directions. This approach can directly capture high-spatial-resolution images from even naturally densely distributed samples such as cell adhesions, whereas it is usually accompanied by a tradeoff in terms of temporal resolution.

*SPSM.* The scattering of surface plasmon waves was first studied in the 1970s [108], and several groups have employed it as the sensing channel to provide more information about the samples in addition to classical SPRM detection [29,109]. SPSM is, however, a novel and promising approach, first appearing in 2020 [88], when it was found to be able to provide high-spatial-resolution SPR images directly and allow high incident intensity even when using commercial industrial CMOS cameras. Furthermore, SPSM can utilize the interference between the surface plasmon waves scattered by the analytes and surface roughness to greatly increase the image intensities of small objects, thus realizing label-free SPR imaging of single proteins in real time for the first time.

**Figure 2:**
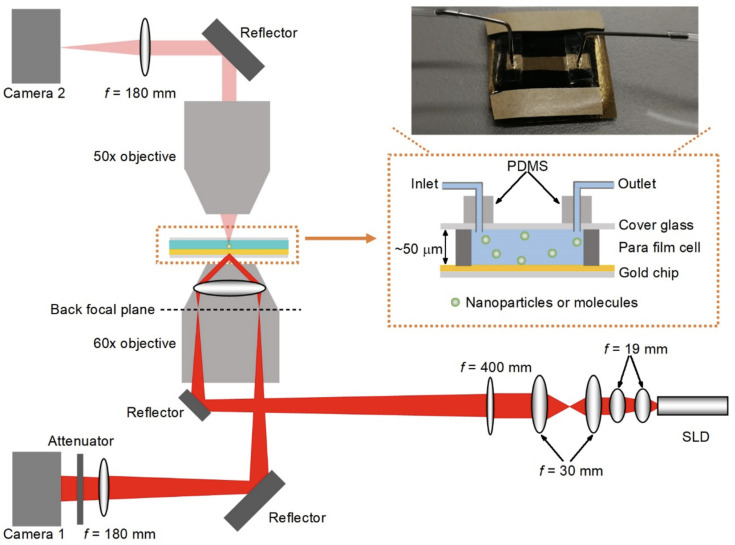
Schematic diagram of the optical setup for simultaneous SPSM and SPRM imaging. Reprinted from Ref. [88] with permission. Copyright 2020 Springer Nature.

SPSM can be constructed easily by adding one objective on the top of either a prism-coupled SPRi system [92] or an SPRM [88] to collect the surface plasmonic waves scattered by the analytes (Figure 2). The flow cell used in SPSM is similar to that used in prism-type total internal reflection fluorescence microscopy, where a cover glass seals the top of the flow cell for optical observation, and the height of the flow channel should be controlled at a few tens of micrometers for easy correction of spherical aberration. SPSM images can be captured simultaneously along with the detector used in the prism-coupled SPRi system or SPRM. As expected, SPSM can directly provide images of single nanoparticles that are free of parabolic tails, reaching higher spatial resolution than SPRM.

For large analytes, scattered surface plasmonic waves dominate SPSM image intensity, while for small analytes, such as polystyrene nanoparticles with diameters smaller than 65 nm, the surface plasmonic waves scattered due to thermally evaporated gold surface roughness will dominate the image intensities, making the analyte intensity change against the volumes. It should be noted that the transition points for pure scattering changing to interference differ with the surface roughness of the gold film. For rougher gold films, the interference may even dominate the SPSM image intensities of polystyrene nanoparticles with diameters larger than 65 nm [88].

## 3. Molecular Interaction Analysis

Determining molecular interaction kinetics is the classical application of SPR techniques. The main advantage of prism-coupled SPRi systems over conventional SPR sensors is their high throughput, where multiple sensing regions with different modifications can be arranged owing to the spatially resolved capabilities, thus allowing for the recognition of multiple components in one sample simultaneously, which is suitable for highly efficient biomarker screening [110,111]. Merging the SPRi method with orthogonal signal amplification enables a direct determination of sub-fM miRNA-15a (a multiple tumor diagnostic biomarker) and is applicable to the direct determination of miRNA-15a in healthy and cancer human serums [112]. Analyses of interactions between platinated DNA and cellular proteins confirmed that SPRi is suitable for better understanding the activity/inactivity mechanisms of drugs and drug screening/discovery [111,113]. In addition, thermodynamic characterization of molecular interactions could also be easily evaluated based on the fitting of temperature-dependent measurements [113,114].

**Figure 3:**
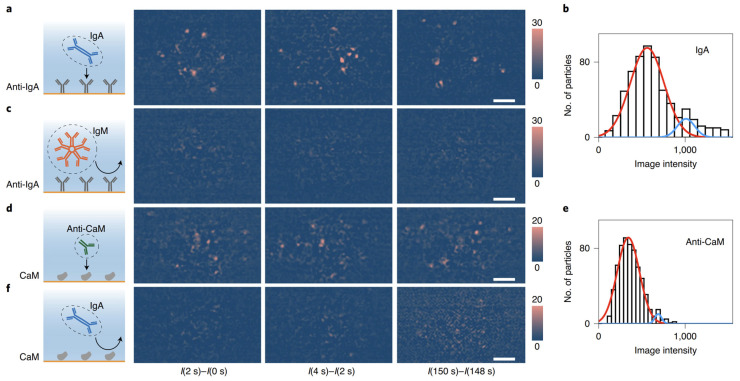
SPSM identification of single proteins using antibodies. Differential snapshots showing binding of IgA (immunoglobulin A) to anti-IgA (**a**), anti-CaM (calmodulin) to CaM (**d**), and corresponding negative control experiment (**c**,**f**); and intensity histogram of IgA molecules (**b**) and anti-CaM molecules (**e**). IgM: immunoglobulin M. Reprinted from Ref. [88] with permission. Copyright 2020 Springer Nature.

**Figure 4:**
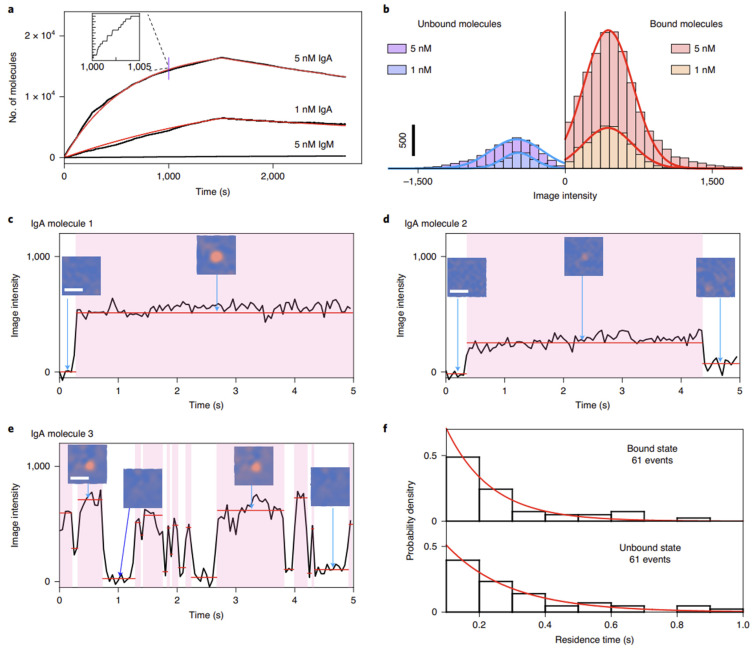
Single-molecule measurement of binding kinetics with SPSM. (**a**) Kinetics of IgA (immunoglobulin A) binding to anti-IgA determined via digital counting of the binding/unbinding of single molecules. (**b**) Histograms of intensity changes associated with binding and unbinding of individual IgA molecules. (**c**–**e**) Examples of different binding behaviors observed at the single-molecule level: IgA molecules 1 (**c**), 2 (**d**) and 3 (**e**). Scale bars of the PSM images, 2 µm. (**f**) Bound and unbound residence time distributions for IgA molecule 3. Reprinted from Ref. [88] with permission. Copyright 2020 Springer Nature.

Based on the single-molecule imaging capabilities, SPSM can recognize and digitally count the single protein binding events over time (Figure 3) and then produce the binding curves for interaction kinetics analysis (Figure 4) [88]. This approach is free of the interferences of bulk refractive index changes and efficiently suppresses the noise resulting from the binding of impurities by analyzing the resident time and analyte mass, thus allowing direct molecular interaction analysis in complex buffers, such as serum [89].

## 4. Molecular Imaging and Profiling

**Figure 5:**
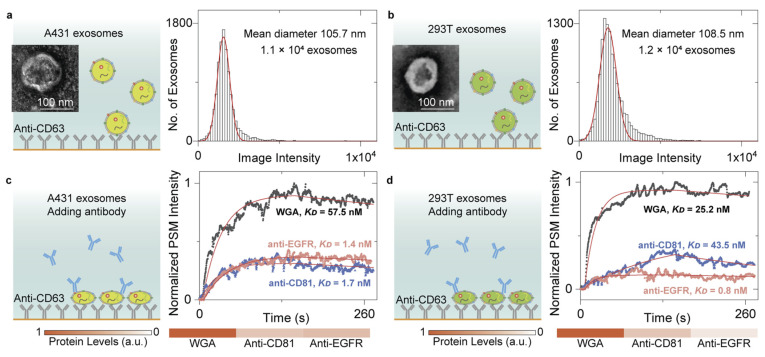
(**a**,**b**) TEM images of extracellular vesicles from A431 (**a**) and 293T (**b**) cells and SPSM image intensity histograms of the exosomes by individually counting the single-binding events. (**c**,**d**) Ensemble SPSM measurements of WGA (wheat germ agglutinin), anti-CD81, and anti-EGFR (epidermal growth factor receptor) binding to the target proteins on the surfaces of A431 (**c**), and 293T (**d**) exosomes. CD63 and CD81 are commonly used exosome surface protein markers. Reprinted from Ref. [91] with permission from the Royal Society of Chemistry.

Owing to the spatially resolved capabilities, prism-coupled SPRi can analyze the spatial distributions of molecular components, enabling molecular imaging and profiling. One classical application of prism-coupled SPRi is that it can image the patterns of fingerprints with adjustable sensitivity and clarity based on a chemically selective stepwise signal amplification strategy [115] and perform quantitative analysis of either glucose or amino acid or both to study metabolic secretions in fingerprints during running exercises [116]. Owing to improved spatial resolution, SPRM and SPSM can further recognize the binding events of single biological entities, such as extracellular vesicles, and then use the antibodies to analyze the relative contents of biomarkers to achieve molecular profiles at the single-entity level (Figure 5) [52,77,91].

## 5. Tracking of Single Entities

Surface plasmon waves on the electric field, with their intensity decreasing exponentially with distance away from the sensor surface, naturally enable high-precision tracking of single-entity motions in the vertical direction (z-direction). However, the average over multiple events in the ensemble SPR system easily blocks the motion details of single entities, whereas the SPRi system can effectively recognize the details of single-entity motions.

Prism and objective SPRi systems have been used to track the movements of single gold nanoparticles modified with target molecules [44,117], which will show different kinds of movements on receptor-modified surfaces due to heterogeneous interactions between the target molecules and receptors. One may calculate the number of target molecules on one nanoparticle by analyzing their free energy profiles, providing a way to sense single molecules. A recent study also showed that the nanoparticles have different resident times on the sensor surface, which is related to the molecular binding kinetics, and thus, one can achieve the binding kinetics profiles by statistically analyzing the resident time of nanoparticles for evaluating the target molecular concentrations in the buffer and clinical samples [44,46,118].

Objective SPRi or SPRM can push the detection limit down to the single-virion level and thus further expand the applications of SPRi to the study of biological entities. In fact, SPRM can track the movements of bacteria during antibiotic processing, providing an in situ approach to evaluating antibiotic resistance [119]. In addition, SPRM can also be used to quantify the movement properties of bacteria captured on the surface with different modifications, paving the road to screening anti-bacterial surface chemical modifications [58].

**Figure 6:**
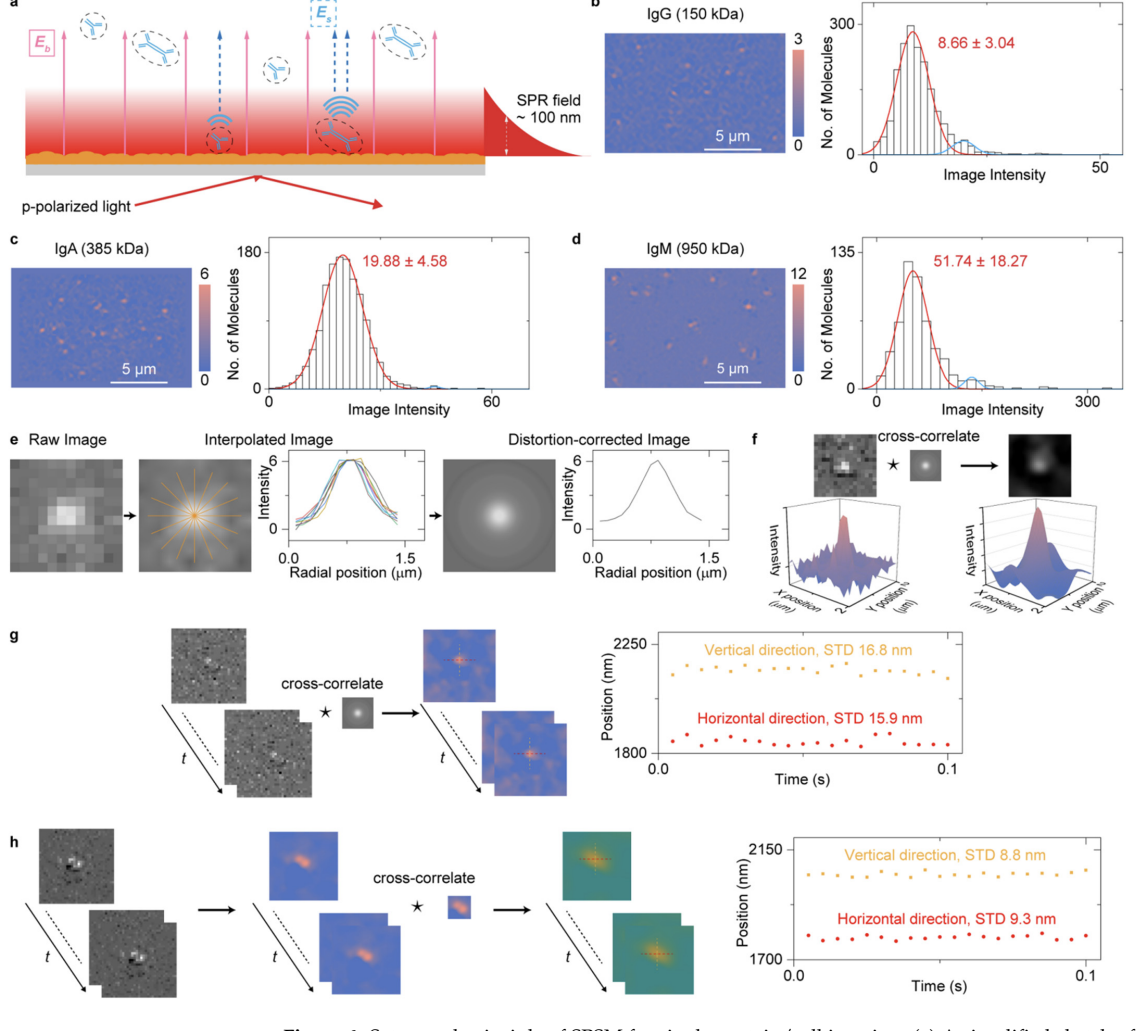
Setup and principle of SPSM for single-protein/cell imaging. (**a**) A simplified sketch of the optical setup. (**b**–**d**) SPSM images and image intensity histograms of IgG (immunoglobulin G, 150 kDa, (**b**)), IgA (immunoglobulin A, 385 kDa, (**c**)), and IgM (immunoglobulin M, 950 kDa, (**d**)) proteins, respectively. (**e**) Extracting mean radial profiles from one differential frame. (**f**) Correcting SPSM image distortions by cross-correlating the SPSM images with the point spread function. (**g**) Tracking the position of one protein binding on the sensor surface. (**h**) Tracking the position of two proteins binding to the nearby surface. Reprinted with permission from Ref. [90] Copyright 2021 American Chemical Society.

In addition, SPSM provides a Gaussian-type point spread function without parabolic tails, which allows not only a high spatial resolution but also the nanometer lateral localization capabilities using the Gaussian fitting. Combined with the single-molecule imaging ability, SPSM can track the movement details of single proteins captured by their antibodies, revealing their heterogeneity during protein interactions in lateral diffusion and free energy properties after statistical analysis at the single-molecule level (Figure 6) [90].

## 6. Analysis of Single Cells

Surface plasmon waves are concentrated within ~100 nm from the sensor surface, thus providing an excellent solution for in situ analysis of ligand interactions with the membrane proteins. A prism-coupled SPRi can spatially resolve the single cells, enabling analysis of the membrane protein content level and binding kinetics with a single-cell resolution [41]. SPRM has further pushed membrane protein researchers to subcellular levels by providing high spatial resolution in the direction perpendicular to the sensing surface [21,23]. 

SPRM with azimuthal rotation illumination can image cell–substrate interactions at the single-adhesion-site level, providing the capabilities for analyzing the cell morphology in detail, whereas it is usually accomplished with tradeoffs in terms of temporal resolution [120].

**Figure 7:**
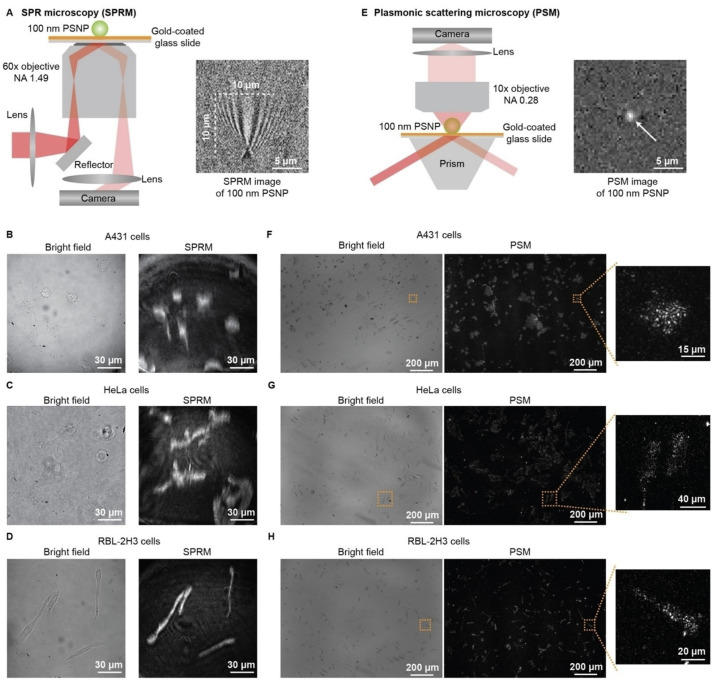
Comparison of SPRM with SPSM imaging of fixed cells. (**A**) Simplified sketch of the optical setup for SPRM and SPRM image of one 100 nm polystyrene nanoparticle (PSNP). (**B**–**D**) Bright-field and SPRM images of fixed A431, HeLa, and RBL-2H3 cells. (**E**) Simplified sketch of the optical setup for PSM and SPSM image of one 100 nm PSNP. (**F**–**H**) Bright-field and SPSM images of fixed A431, HeLa, and RBL-2H3 cells. Reproduced with permission from Ref. [92] Copyright 2022 Wiley VCH.

**Figure 8:**
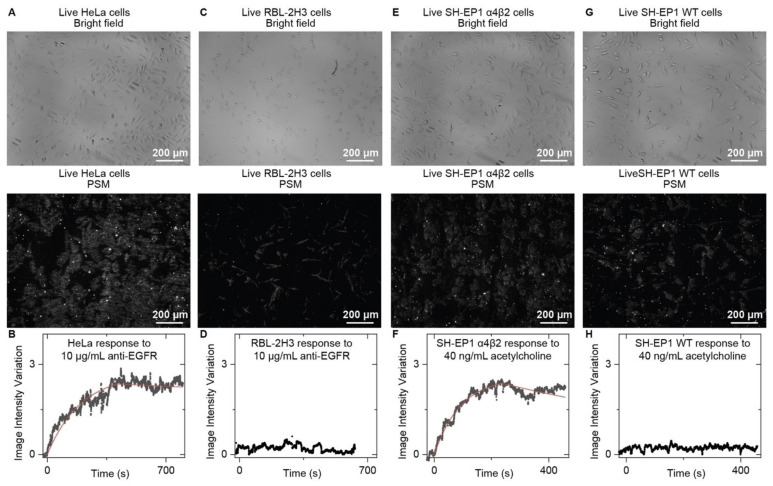
SPSM measurement of antibody and small molecule binding kinetics on live cells. (**A**) Bright-field and SPSM images of live HeLa cells. (**B**) The image intensity variation against time by averaging the signals of all cells within the field of view shown in (**A**) during the anti-EGFR (epidermal growth factor receptor) binding process. (**C**) Bright-field and SPSM images of live RBL-2H3 Cells. (**D**) The image intensity variation against time by averaging the signal of all cells within the field of view shown in (**C**) during exposure to anti-EGFR as the negative control. (**E**) Bright-field and SPSM images of live SH-EP1 α4β2 Cells. (**F**) The image intensity variation against time by averaging the signals of all cells within the field of view shown in (**E**) during the acetylcholine binding process. (**G**) Bright-field and SPSM images of live SH-EP1 WT Cells. (**H**) The image intensity variation against time by averaging the signal of all cells within the field of view shown in (**G**) during exposure to acetylcholine as the negative control. Reproduced with permission from Ref. [92] Copyright 2022 Wiley VCH.

SPSM can image the cell adhesion sites in real time, which not only allows for analyzing membrane protein binding kinetics at the single-cell level but also the movement analysis of single adhesion sites in detail, revealing that heterogeneity also exists among the adhesion sites even for one cell. Furthermore, SPSM can be constructed in a prism-coupled SPRi system, where a large illumination area can be easily achieved by using a large prism. Combined with a large view camera, SPSM has been demonstrated to be able to provide a millimeter-scale field of view for in situ analysis of hundreds of cells at the single-adhesion-site level, which is usually challenging for SPRM due to the performance limit of objective (Figure 7 and Figure 8) [92].

## 7. Development of New Methods

*Evanescent scattering microscopy*. For optical imaging approaches, including SPRi, spatial resolution is fundamentally limited by optical diffraction. Except for using high numerical aperture objectives, the other effective way is using short illumination wavelengths. However, SPRi usually uses a gold film to excite the surface plasmon waves, which is usually only available for incident wavelengths longer than 530 nm. Recently developed evanescent scattering microscopy [121] removed the gold film and directly used evanescent waves excited by total internal reflection as the illumination field. The evanescent waves have similar properties to the surface plasmon waves, so evanescent scattering microscopy can achieve similar image features in SPSM with higher spatial resolution owing to the employment of blue incident light. However, the evanescent waves have lower electric field enhancements than surface plasmon waves, limiting further improvement in terms of signal–noise ratio. Nevertheless, this method still demonstrates one way to further improve the spatial resolution of SPRi by employing short incident wavelengths, which may rely on the new materials supporting surface plasmon waves with short incident wavelengths.

*Multi-parameter analysis.* It is well known that biological entities, including cells, extracellular vesicles, and molecules, are highly heterogeneous, demanding quantitative analysis approaches. SPRi approaches detect the refractive index changes near the surface, which is related to analyte mass information. In addition, SPRi can also be combined with other techniques for multi-parameter analysis. For example, an electric field can be applied onto the gold surface, and the analyte movement forced by the electric field can be monitored via SPRi with high precision in the vertical direction owing to the exponential decaying properties of surface plasmon waves, thus allowing for quantitative analysis of analyte electric properties, such as charge and mobility [122]. SPRi can also be combined with fluorescence imaging techniques, while SPRi can track the variations in cellular membranes with high temporal resolution, and fluorescence imaging can provide additional information, such as calcium ion concentrations inside the cells [123]. These approaches demonstrate that SPRi is a great platform for a combination of multiple approaches for multi-parameter analysis to understand biological processes from different aspects.

## 8. Summary and Outlook

SPRi technology has pushed beyond ensemble averages and revealed the statistical distributions of biological entities and processes while maintaining the label-free and high-sensitivity advantages of traditional SPR techniques. This advance mainly results from the spatially resolved capabilities provided by SPRi. SPRM has demonstrated that using a high numerical aperture objective can help improve the spatial resolution of SPRi and other kinds of optical imaging approaches. Furthermore, SPSM demonstrates that the scattering detection scheme can effectively suppress the effects of delocalized propagation properties of surface plasmon waves along the surface to directly achieve diffraction-limited spatial resolution (Table 1).

Spatial resolution enhancement remains a topic requiring further studies as well as detection sensitivity. Other advanced optical imaging approaches, such as interferometric scattering microscopy, have demonstrated that short wavelengths can provide larger scattering sections for enhancing the signal intensity to improve detection sensitivity and also permit high spatial resolution according to the Rayleigh criterion without varying the optical arrangements of the systems [66,124,125,126,127,128,129,130,131,132,133,134,135,136,137]. However, this approach is hard to realize using gold-coated glass slides, which are commonly employed in current SPR systems and only permit the excitation of surface plasmon waves with incident wavelengths longer than 530 nm. To overcome this limitation, a promising solution is developing new sensing structures using silver or aluminum as the layer supporting surface plasmonic waves with short incident wavelengths, such as blue or green lights. At the same time, physical or chemical modifications on the sensor surfaces are also required to protect the sensing structures from the oxidation effect. In addition, novel super-resolution localization approaches should also be developed in combination with high-spatial-resolution SPRi systems to build novel label-free super-resolution imaging approaches. After establishing the new high-spatial-resolution SPRi, its applications can be expanded to multi-parameter analysis, except for traditional molecular imaging applications, which may provide a powerful tool for understanding biological processes from different aspects.

## Figures and Tables

**Table 1:** Comparison of several imaging methods.

Imaging Method	Spatial Resolution	Field of View	Sensitivity	Time Resolution	Applications	Advantage	Disadvantage
SPRi based on prism	micrometer level, limited by long propagation length of surface plasmonic wave.	millimeter scale	low	limited by speed of the imager	multiple molecule events in ensemble, single cell	simple and low-cost device	low spatial resolution
SPRM based on high numerical aperture objective	sub-micrometer level, limited by diffraction effect perpendicular to propagation direction of surface plasmonic waves; micrometer level, limited by propagation length of surface plasmonic wave along the propagation direction of surface plasmonic wave.	small, ~160 μm in diameter, limited by high numerical aperture objective	low		single biological entities such as cell (subcellular level) and bacterial, sparsely distributed single nanoparticles, etc. single-molecule sensing by monitoring change in *z*-axis fluctuations of nanoparticle modified with target molecules.	improved spatial resolution	SPRM images of the analyte are accompanied by a parabolic tail of many microns long. (SPRM with azimuthal rotation illumination can improve resolution with tradeoff of temporal resolution)
SPSM	sub-micrometer or micrometer level, diffraction-limited at any lateral direction. For NA 0.28 of imaging objective, spatial resolution can be ~1.4 μm, which is ~7 times higher than SPRM with same wavelength of 660 nm.	small, ~160 μm in diameter, for objective-coupled SPSM; millimeter scale for prism-coupled SPSM.	large proteins as small as IgG (150 kDa) [121]		single molecules, single biological entities such as cell (at single adhesion site level) and exosome, densely distributed single nanoparticles, etc. simultaneous analyses of hundreds of cells by prism-coupled SPSM.	high spatial resolution with Gaussian point spread function without parabolic tails, large field of view for prism-coupled SPSM	
Evanescent scattering microscopy (ESM)	sub-micrometer or micrometer level, diffraction-limited at any lateral direction, with higher spatial resolution than SPSM owing employment of shorter incident wavelength.	small, ~160 μm in diameter, for objective-coupled ESM; millimeter scale for prism-coupled ESM.	medium-sized single proteins as small as BSA (66 kDa) [121]		single molecules, single biological entities such as cell ( at single adhesion site level) and exosome, densely distributed single nanoparticles, etc. simultaneous in situ analyses of hundreds of cells by prism-coupled ESM.	higher spatial resolution owing the employment of short incident wavelength than SPSM.	the evanescent waves have lower electric field enhancements than surface plasmon waves.

## Data Availability

Not applicable.

## References

[1] Copeland R.A., Pompliano D.L., Meek T.D. (2006). Drug–target residence time and its implications for lead optimization. Nat. Rev. Drug Discov..

[2] Robers M.B., Dart M.L., Woodroofe C.C., Zimprich C.A., Kirkland T.A., Machleidt T., Kupcho K.R., Levin S., Hartnett J.R., Zimmerman K. (2015). Target engagement and drug residence time can be observed in living cells with BRET. Nat. Commun..

[3] Copeland R.A. (2016). The drug–target residence time model: A 10-year retrospective. Nat. Rev. Drug Discov..

[4] de Witte W.E.A., Danhof M., van der Graaf P.H., de Lange E.C.M. (2019). The implications of target saturation for the use of drug–target residence time. Nat. Rev. Drug Discov..

[5] Bernetti M., Masetti M., Rocchia W., Cavalli A. (2019). Kinetics of Drug Binding and Residence Time. Annu. Rev. Phys. Chem..

[6] Pantsar T., Kaiser P.D., Kudolo M., Forster M., Rothbauer U., Laufer S.A. (2022). Decisive role of water and protein dynamics in residence time of p38α MAP kinase inhibitors. Nat. Commun..

[7] Homola J., Yee S., Gauglitz G. (1999). Surface plasmon resonance sensors: Review. Sens. Actuators B-Chem..

[8] Arroyo J.O., Kukura P. (2016). Non-fluorescent schemes for single-molecule detection, imaging and spectroscopy. Nat. Photonics.

[9] Tao N.J., Boussaad S., Huang W.L., Arechabaleta R.A., D’Agnese J. (1999). High resolution surface plasmon resonance spectroscopy. Rev. Sci. Instrum..

[10] Boussaad S., Pean J., Tao N.J. (2000). High-Resolution Multiwavelength Surface Plasmon Resonance Spectroscopy for Probing Conformational and Electronic Changes in Redox Proteins. Anal. Chem..

[11] Wang S., Boussaad S., Wong S., Tao N.J. (2000). High-Sensitivity Stark Spectroscopy Obtained by Surface Plasmon Resonance Measurement. Anal. Chem..

[12] Wang S., Boussaad S., Tao N.J. (2001). Surface plasmon resonance enhanced optical absorption spectroscopy for studying molecular adsorbates. Rev. Sci. Instrum..

[13] Zhang H.Q., Boussaad S., Tao N.J. (2003). High-performance differential surface plasmon resonance sensor using quadrant cell photodetector. Rev. Sci. Instrum..

[14] Homola J. (2003). Present and future of surface plasmon resonance biosensors. Anal. Bioanal. Chem..

[15] Forzani E.S., Zhang H., Chen W., Tao N. (2005). Detection of Heavy Metal Ions in Drinking Water Using a High-Resolution Differential Surface Plasmon Resonance Sensor. Environ. Sci. Technol..

[16] Wang S., Forzani E.S., Tao N. (2007). Detection of Heavy Metal Ions in Water by High-Resolution Surface Plasmon Resonance Spectroscopy Combined with Anodic Stripping Voltammetry. Anal. Chem..

[17] Shan X., Foley K.J., Tao N. (2008). A label-free optical detection method for biosensors and microfluidics. Appl. Phys. Lett..

[18] Homola J. (2008). Surface Plasmon Resonance Sensors for Detection of Chemical and Biological Species. Chem. Rev..

[19] Shan X., Huang X., Foley K.J., Zhang P., Chen K., Wang S., Tao N. (2010). Measuring Surface Charge Density and Particle Height Using Surface Plasmon Resonance Technique. Anal. Chem..

[20] Shan X., Patel U., Wang S., Iglesias R., Tao N. (2010). Imaging Local Electrochemical Current via Surface Plasmon Resonance. Science.

[21] Wang W., Foley K., Shan X., Wang S., Eaton S., Nagaraj V.J., Wiktor P., Patel U., Tao N. (2011). Single cells and intracellular processes studied by a plasmonic-based electrochemical impedance microscopy. Nat. Chem..

[22] Mayer K.M., Hafner J.H. (2011). Localized Surface Plasmon Resonance Sensors. Chem. Rev..

[23] Wang W., Yang Y., Wang S., Nagaraj V.J., Liu Q., Wu J., Tao N. (2012). Label-free measuring and mapping of binding kinetics of membrane proteins in single living cells. Nat. Chem..

[24] Guo X. (2012). Surface plasmon resonance based biosensor technique: A review. J. Biophotonics.

[25] Mariani S., Minunni M. (2014). Surface plasmon resonance applications in clinical analysis. Anal. Bioanal. Chem..

[26] Wong C.L., Olivo M. (2014). Surface Plasmon Resonance Imaging Sensors: A Review. Plasmonics.

[27] Lee T.-H., Hirst D.J., Kulkarni K., Del Borgo M.P., Aguilar M.-I. (2018). Exploring Molecular-Biomembrane Interactions with Surface Plasmon Resonance and Dual Polarization Interferometry Technology: Expanding the Spotlight onto Biomembrane Structure. Chem. Rev..

[28] Yeatman E., Ash E. (1987). Surface-Plasmon Microscopy. Electron. Lett..

[29] Rothenhausler B., Knoll W. (1988). Surface-Plasmon Microscopy. Nature.

[30] Liu L., He Y., Zhang Y., Ma S., Ma H., Guo J. (2008). Parallel scan spectral surface plasmon resonance imaging. Appl. Opt..

[31] Liu Z., Ma S., Ji Y., Liu L., Hu Z., Guo J., Ma H., He Y. (2010). Quasi-Confocal, Multichannel Parallel Scan Hyperspectral Fluorescence Imaging Method Optimized for Analysis of Multicolor Microarrays. Anal. Chem..

[32] Liu Z., He Y., Liu L., Ma S., Chong X., Hu Z., Ma H., Guo J. (2010). Two-channel, quasi-confocal parallel scan fluorescence imaging for detection of biochips. Opt. Lasers Eng..

[33] Liu Z., Yang L., Liu L., Chong X., Guo J., Ma S., Ji Y., He Y. (2011). Parallel-scan based microarray imager capable of simultaneous surface plasmon resonance and hyperspectral fluorescence imaging. Biosens. Bioelectron..

[34] Liu L., Ma S., Ji Y., Chong X., Liu Z., He Y., Guo J. (2011). A two-dimensional polarization interferometry based parallel scan angular surface plasmon resonance biosensor. Rev. Sci. Instrum..

[35] Liu L., Chen X., Liu Z., Ma S., Du C., He Y., Guo J. (2012). Polarization interference interrogation of angular surface plasmon resonance sensors with wide metal film thickness tolerance. Sens. Actuators B Chem..

[36] Shi H., Liu Z., Wang X., Guo J., Liu L., Luo L., Guo J., Ma H., Sun S., He Y. (2013). A symmetrical optical waveguide based surface plasmon resonance biosensing system. Sens. Actuators B Chem..

[37] Jing W., Wang Y., Yang Y., Wang Y., Ma G., Wang S., Tao N. (2019). Time-Resolved Digital Immunoassay for Rapid and Sensitive Quantitation of Procalcitonin with Plasmonic Imaging. ACS Nano.

[38] Berger C., Kooyman R., Greve J. (1994). Resolution in Surface-Plasmon Microscopy. Rev. Sci. Instrum..

[39] Yin L., Wang W., Wang S., Zhang F., Zhang S., Tao N. (2015). How does fluorescent labeling affect the binding kinetics of proteins with intact cells?. Biosens. Bioelectron..

[40] Yin L., Yang Y., Wang S., Wang W., Zhang S., Tao N. (2015). Measuring Binding Kinetics of Antibody-Conjugated Gold Nanoparticles with Intact Cells. Small.

[41] Zhang F., Wang S., Yin L., Yang Y., Guan Y., Wang W., Xu H., Tao N. (2015). Quantification of Epidermal Growth Factor Receptor Expression Level and Binding Kinetics on Cell Surfaces by Surface Plasmon Resonance Imaging. Anal. Chem..

[42] Huang B., Yu F., Zare R. (2007). Surface plasmon resonance imaging using a high numerical aperture microscope objective. Anal. Chem..

[43] Yu H., Shan X., Wang S., Chen H., Tao N. (2014). Plasmonic Imaging and Detection of Single DNA Molecules. ACS Nano.

[44] Wang H., Tang Z., Wang Y., Ma G., Tao N. (2019). Probing Single Molecule Binding and Free Energy Profile with Plasmonic Imaging of Nanoparticles. J. Am. Chem. Soc..

[45] Ma G., Wan Z., Zhu H., Tao N. (2020). Roles of entropic and solvent damping forces in the dynamics of polymer tethered nanoparticles and implications for single molecule sensing. Chem. Sci..

[46] Wang Y., Tang Z., Chen H.-Y., Wang W., Tao N., Wang H. (2021). Single-molecule calorimeter and free energy landscape. Proc. Natl. Acad. Sci. USA.

[47] Yang Y., Yu H., Shan X., Wang W., Liu X., Wang S., Tao N. (2015). Label-Free Tracking of Single Organelle Transportation in Cells with Nanometer Precision Using a Plasmonic Imaging Technique. Small.

[48] Ma G., Syu G.-D., Shan X., Henson B., Wang S., Desai P.J., Zhu H., Tao N. (2018). Measuring Ligand Binding Kinetics to Membrane Proteins Using Virion Nano-oscillators. J. Am. Chem. Soc..

[49] Wang S., Shan X., Patel U., Huang X., Lu J., Li J., Tao N. (2010). Label-free imaging, detection, and mass measurement of single viruses by surface plasmon resonance. Proc. Natl. Acad. Sci. USA.

[50] Liu R., Shan X., Wang H., Tao N. (2019). Plasmonic Measurement of Electron Transfer between a Single Metal Nanoparticle and an Electrode through a Molecular Layer. J. Am. Chem. Soc..

[51] Wang H., Yu H., Wang Y., Shan X., Chen H.-Y., Tao N. (2020). Phase imaging of transition from classical to quantum plasmonic couplings between a metal nanoparticle and a metal surface. Proc. Natl. Acad. Sci. USA.

[52] Yang Y., Shen G., Wang H., Li H., Zhang T., Tao N., Ding X., Yu H. (2018). Interferometric plasmonic imaging and detection of single exosomes. Proc. Natl. Acad. Sci. USA.

[53] Qian C., Wu G., Jiang D., Zhao X., Chen H.-B., Yang Y., Liu X.-W. (2019). Identification of Nanoparticles via Plasmonic Scattering Interferometry. Angew. Chem. Int. Ed..

[54] Liu Y.-N., Chen H.-B., Liu X.-W. (2020). Rapid Assessment of Water Toxicity by Plasmonic Nanomechanical Sensing. Anal. Chem..

[55] Zhou X.-L., Yang Y., Wang S., Liu X.-W. (2020). Surface Plasmon Resonance Microscopy: From Single-Molecule Sensing to Single-Cell Imaging. Angew. Chem. Int. Ed..

[56] Chen H.-B., Jiang D., Liu Y.-N., Qian C., Zhou X.-L., Liu X.-W. (2020). Probing the Deposition Kinetics of Nanoparticles by Plasmonic Imaging and Counting Single Nanoparticles. Environ. Sci. Technol. Lett..

[57] Chen H.-B., Jiang D., Zhou X.-L., Qian C., Yang Y., Liu X.-W. (2020). Tracking Interfacial Dynamics of a Single Nanoparticle Using Plasmonic Scattering Interferometry. Anal. Chem..

[58] Liu Y.-N., Lv Z.-T., Lv W.-L., Liu X.-W. (2020). Plasmonic probing of the adhesion strength of single microbial cells. Proc. Natl. Acad. Sci. USA.

[59] Jiang D., Chen H.-B., Qian C., Zhou X.-L., Liu X.-W. (2021). Determining the Aggregation Kinetics of Nanoparticles by Single Nanoparticle Counting. ACS EST Water.

[60] Liu Y.-N., Lv Z.-T., Yang S.-Y., Liu X.-W. (2021). Optical Tracking of the Interfacial Dynamics of Single SARS-CoV-2 Pseudoviruses. Environ. Sci. Technol..

[61] Zhou X., Hao H., Zhang Y.-J., Zheng Q., Tan S., Zhao J., Chen H.-B., Chen J.-J., Gu Y., Yu H.-Q. (2021). Patterning of transition metal dichalcogenides catalyzed by surface plasmons with atomic precision. Chem.

[62] Jiang D., Zhao X., Liu Y.-N., Chen H.-B., Lv W.-L., Qian C., Liu X.-W. (2021). Label-Free Probing of Molecule Binding Kinetics Using Single-Particle Interferometric Imaging. Anal. Chem..

[63] Jiang D., Chen H.-B., Zhou X.-L., Liu X.-W. (2022). Single-Particle Electrochemical Imaging Provides Insights into Silver Nanoparticle Dissolution at the Solution–Solid Interface. ACS Appl. Mater. Interfaces.

[64] Lv Z.-T., Qian C., Liu Y.-N., Lv Y.-H., Liu X.-W. (2022). Optical Tracking of Surfactant-Tuned Bacterial Adhesion: A Single-Cell Imaging Study. Appl. Environ. Microbiol..

[65] Zhao X., Zhou X.-L., Yang S.-Y., Min Y., Chen J.-J., Liu X.-W. (2022). Plasmonic imaging of the layer-dependent electrocatalytic activity of two-dimensional catalysts. Nat. Commun..

[66] Wu G., Qian C., Lv W., Zhao X., Liu X. (2023). Dynamic imaging of interfacial electrochemistry on single Ag nanowires by azimuth-modulated plasmonic scattering interferometry. Nat. Commun..

[67] Liu Y.-N., Lv Z.-T., Lv W.-L., Liu D.-F., Liu X.-W. (2023). Label-Free Optical Imaging of the Electron Transfer in Single Live Microbial Cells. Nano Lett..

[68] Wan J.-H., Qian C., Wu G., Liu X.-W. (2023). High-Resolution, High-Throughput Plasmonic Imaging of Nanomaterials. Anal. Chem..

[69] Lv W.-L., Qian C., Cao C.-X., Lv Z.-T., Liu X.-W. (2023). Plasmonic Scattering Imaging of Surface-Bonded Nanoparticles at the Solution–Solid Interface. ACS Appl. Mater. Interfaces.

[70] Wang Z.-K., Yuan Z.-X., Qian C., Liu X.-W. (2023). Plasmonic Probing of Deoxyribonucleic Acid Hybridization at the Single Base Pair Resolution. Anal. Chem..

[71] Jiang Y., Su H., Wei W., Wang Y., Chen H.-Y., Wang W. (2019). Tracking the rotation of single CdS nanorods during photocatalysis with surface plasmon resonance microscopy. Proc. Natl. Acad. Sci. USA.

[72] Liu S., Zhou K., Yuan T., Lei W., Chen H.-Y., Wang X., Wang W. (2020). Imaging the Thermal Hysteresis of Single Spin-Crossover Nanoparticles. J. Am. Chem. Soc..

[73] Wang Y., Yuan T., Su H., Zhou K., Yin L., Wang W. (2021). A Bubble-STORM Approach for Super-Resolved Imaging of Nucleation Sites in Hydrogen Evolution Reactions. ACS Sens..

[74] Yu H., Shan X., Wang S., Chen H., Tao N. (2014). Molecular Scale Origin of Surface Plasmon Resonance Biosensors. Anal. Chem..

[75] Yu H., Shan X., Wang S., Tao N. (2017). Achieving High Spatial Resolution Surface Plasmon Resonance Microscopy with Image Reconstruction. Anal. Chem..

[76] Yang Y., Zhai C., Zeng Q., Khan A.L., Yu H. (2019). Quantitative Amplitude and Phase Imaging with Interferometric Plasmonic Microscopy. ACS Nano.

[77] Yang Y., Zhai C., Zeng Q., Khan A., Yu H. (2020). Multifunctional Detection of Extracellular Vesicles with Surface Plasmon Resonance Microscopy. Anal. Chem..

[78] Xu Y., Li C., Jiang Y., Guo M., Yang Y., Yang Y., Yu H. (2020). Electrochemical Impedance Spectroscopic Detection of *E. coli* with Machine Learning. J. Electrochem. Soc..

[79] Wang X., Zeng Q., Xie F., Wang J., Yang Y., Xu Y., Li J., Yu H. (2021). Automated Nanoparticle Analysis in Surface Plasmon Resonance Microscopy. Anal. Chem..

[80] Xu Y., Wang X., Zhai C., Wang J., Zeng Q., Yang Y., Yu H. (2021). A Single-Shot Autofocus Approach for Surface Plasmon Resonance Microscopy. Anal. Chem..

[81] Yang Y., Zeng Q., Luo Q., Wang C., Yu H. (2022). Dynamic Single-Molecule Sensors: A Theoretical Study. ACS Sens..

[82] Zeng Q., Zhou X., Yang Y., Sun Y., Wang J., Zhai C., Li J., Yu H. (2022). Dynamic single-molecule sensing by actively tuning binding kinetics for ultrasensitive biomarker detection. Proc. Natl. Acad. Sci. USA.

[83] Zhai C., Xie F., Xu J., Yang Y., Zheng W., Hu H., Ding X., Yu H. (2023). Correlation between membrane proteins and sizes of extracellular vesicles and particles: A potential signature for cancer diagnosis. J. Extracell. Vesicles.

[84] Sun Y., Zhang C., Yang Y.-t., Yu H., Li J.-H. (2023). Polarization-Sensitive Asymmetric Scattering at the Single-Particle Scale via Surface Plasmon Resonance Microscopy. Anal. Chem..

[85] Zhai C., Long J., He J., Zheng Y., Wang B., Xu J., Yang Y., Jiang L., Yu H., Ding X. (2023). Precise Identification and Profiling of Surface Proteins of Ultra Rare Tumor Specific Extracellular Vesicle with Dynamic Quantitative Plasmonic Imaging. ACS Nano.

[86] Son T., Lee C., Seo J., Choi I.H., Kim D. (2018). Surface plasmon microscopy by spatial light switching for label-free imaging with enhanced resolution. Opt. Lett..

[87] Kuai Y., Chen J., Tang X., Xiang Y., Lu F., Kuang C., Xu L., Shen W., Cheng J., Gui H. (2019). Label-free surface-sensitive photonic microscopy with high spatial resolution using azimuthal rotation illumination. Sci. Adv..

[88] Zhang P., Ma G., Dong W., Wan Z., Wang S., Tao N. (2020). Plasmonic scattering imaging of single proteins and binding kinetics. Nat. Methods.

[89] Zhang P., Ma G., Wan Z., Wang S. (2021). Quantification of Single-Molecule Protein Binding Kinetics in Complex Media with Prism-Coupled Plasmonic Scattering Imaging. ACS Sens..

[90] Zhang P., Zhou X., Wang R., Jiang J., Wan Z., Wang S. (2021). Label-Free Imaging of Nanoscale Displacements and Free-Energy Profiles of Focal Adhesions with Plasmonic Scattering Microscopy. ACS Sens..

[91] Zhang P., Jiang J., Zhou X., Kolay J., Wang R., Wan Z., Wang S. (2022). Label-free imaging and biomarker analysis of exosomes with plasmonic scattering microscopy. Chem. Sci..

[92] Zhang P., Zhou X., Jiang J., Kolay J., Wang R., Ma G., Wan Z., Wang S. (2022). In Situ Analysis of Membrane-Protein Binding Kinetics and Cell–Surface Adhesion Using Plasmonic Scattering Microscopy. Angew. Chem. Int. Ed..

[93] Bocková M., Slaby J., Springer T., Homola J., Bohn P., Pemberton J. (2019). Advances in Surface Plasmon Resonance Imaging and Microscopy and Their Biological Applications. Annu. Rev. Anal. Chem..

[94] Huo Z., Li Y., Chen B., Zhang W., Yang X., Yang X. (2023). Recent advances in surface plasmon resonance imaging and biological applications. Talanta.

[95] Wang Q., Ren Z., Zhao W., Wang L., Yan X., Zhu A., Qiu F., Zhang K. (2022). Research advances on surface plasmon resonance biosensors. Nanoscale.

[96] Huang S.F., Chen J.J., Zhang T.L., Dai X.Q., Wang X.L., Zhou J.X., Kong W.F., Liu Q., Qu J.L., Shao Y.H. (2022). Recent Advances in Surface Plasmon Resonance Microscopy. Chemosensors.

[97] Ding X.X., Cao Y.T., Wang X., Lu X.C., Huang C.J. (2022). Review-Advances in Surface Plasmon Resonance Microscopy and Its Applications to Single Cells, Viruses, and Molecules. J. Electrochem. Soc..

[98] Chin L., Son T., Hong J., Liu A., Skog J., Castro C., Weissleder R., Lee H., Im H. (2020). Plasmonic Sensors for Extracellular Vesicle Analysis: From Scientific Development to Translational Research. ACS Nano.

[99] Kretschm E., Raether H. (1968). Radiative Decay of Non Radiative Surface Plasmons Excited by Light. Z. Naturforschung Part A-Astrophys. Phys. Und Phys. Chem. A.

[100] Beusink J.B., Lokate A.M.C., Besselink G.A.J., Pruijn G.J.M., Schasfoort R.B.M. (2008). Angle-scanning SPR imaging for detection of biomolecular interactions on microarrays. Biosens. Bioelectron..

[101] Otsuki S., Ishikawa M. (2010). Wavelength-scanning surface plasmon resonance imaging for label-free multiplexed protein microarray assay. Biosens Bioelectron.

[102] Shen G., Han Z., Liu W., Chen Y. (2007). Color surface plasmon resonance imaging. Chem. J. Chin. Univ.-Chin..

[103] Xu J., Chen Y. (2016). A Coupling Scheme of Surface Plasmon Resonance Sensing. China Patent.

[104] Xu J., Chen Y. (2016). A Scissor Type Surface Plasmon Resonance Imager Based on Liquid Core Coupler. China Patent.

[105] Xu J., Chen Y. (2017). Surface Plasmon Resonance Imager Based on Liquid Core Coupler. China Patent.

[106] Xu J., Chen Y. (2018). Surface plasmon resonance sensing with adjustable sensitivity based on a flexible liquid core coupling unit. Talanta.

[107] Liu C., Hu F., Yang W., Xu J., Chen Y. (2017). A critical review of advances in surface plasmon resonance imaging sensitivity. TrAC Trends Anal. Chem..

[108] Ritchie R. (1970). Optical Emission from Surface Plasmons. Phys. Status Solidi.

[109] Loison O., Fort E. (2013). Transmission surface plasmon resonance microscopy. Appl. Phys. Lett..

[110] Ladd J., Taylor A., Piliarik M., Homola J., Jiang S. (2009). Label-free detection of cancer biomarker candidates using surface plasmon resonance imaging. Anal. Bioanal. Chem..

[111] Chrastinova L., Pastva O., Bockova M., Sacha P., Suttnar J., Stikarova J., Hlavackova A., Kotlin R., Cermak J., Homola J. (2016). The Potential Prognostic Markers for Myelodysplatic Syndromes Studied By Surface Plasmon Resonance Imaging and Mass Spectrometry. Blood.

[112] Hu F., Xu J., Chen Y. (2017). Surface Plasmon Resonance Imaging Detection of Sub-femtomolar MicroRNA. Anal. Chem..

[113] Wang X., Xu J., Liu C., Chen Y. (2016). Specific interaction of platinated DNA and proteins by surface plasmon resonance imaging. RSC Adv..

[114] Juhász A., Csapó E., Ungor D., Tóth G., Vécsei L., Dékány I. (2016). Kinetic and Thermodynamic Evaluation of Kynurenic Acid Binding to GluR1_270-300_ Polypeptide by Surface Plasmon Resonance Experiments. J. Phys. Chem. B.

[115] Liu C., Wang X., Xu J., Chen Y. (2016). Chemical Strategy to Stepwise Amplification of Signals in Surface Plasmon Resonance Imaging Detection of Saccharides and Glycoconjugates. Anal. Chem..

[116] Li M., Xu J., Zheng Q., Guo C., Chen Y. (2022). Chemical-Based Surface Plasmon Resonance Imaging of Fingerprints. Anal. Chem..

[117] Ma G., Shan X., Wang S., Tao N. (2019). Quantifying Ligand–Protein Binding Kinetics with Self-Assembled Nano-oscillators. Anal. Chem..

[118] Wang Y., Jing W., Tao N., Wang H. (2021). Probing Single-Molecule Binding Event by the Dynamic Counting and Mapping of Individual Nanoparticles. ACS Sens..

[119] Syal K., Iriya R., Yang Y., Yu H., Wang S., Haydel S.E., Chen H.-Y., Tao N. (2016). Antimicrobial Susceptibility Test with Plasmonic Imaging and Tracking of Single Bacterial Motions on Nanometer Scale. ACS Nano.

[120] Son T., Lee C., Moon G., Lee D., Cheong E., Kim D. (2019). Enhanced surface plasmon microscopy based on multi-channel spatial light switching for label-free neuronal imaging. Biosens. Bioelectron..

[121] Zhang P., Zhou L., Wang R., Zhou X., Jiang J., Wan Z., Wang S. (2022). Evanescent scattering imaging of single protein binding kinetics and DNA conformation changes. Nat. Commun..

[122] Shan X., Fang Y., Wang S., Guan Y., Chen H.-Y., Tao N. (2014). Detection of Charges and Molecules with Self-Assembled Nano-Oscillators. Nano Lett..

[123] Wang R., Jiang J., Zhou X., Wan Z., Zhang P., Wang S. (2022). Rapid Regulation of Local Temperature and Transient Receptor Potential Vanilloid 1 Ion Channels with Wide-Field Plasmonic Thermal Microscopy. Anal. Chem..

[124] Piliarik M., Sandoghdar V. (2014). Direct optical sensing of single unlabelled proteins and super-resolution imaging of their binding sites. Nat. Commun..

[125] Ortega Arroyo J., Cole D., Kukura P. (2016). Interferometric scattering microscopy and its combination with single-molecule fluorescence imaging. Nat. Protoc..

[126] Cole D., Young G., Weigel A., Sebesta A., Kukura P. (2017). Label-Free Single-Molecule Imaging with Numerical-Aperture-Shaped Interferometric Scattering Microscopy. ACS Photonics.

[127] Young G., Hundt N., Cole D., Fineberg A., Andrecka J., Tyler A., Olerinyova A., Ansari A., Marklund E.G., Collier M.P. (2018). Quantitative mass imaging of single biological macromolecules. Science.

[128] Park J.-S., Lee I.-B., Moon H.-M., Joo J.-H., Kim K.-H., Hong S.-C., Cho M. (2018). Label-free and live cell imaging by interferometric scattering microscopy. Chem. Sci..

[129] Taylor R.W., Mahmoodabadi R.G., Rauschenberger V., Giessl A., Schambony A., Sandoghdar V. (2019). Interferometric scattering microscopy reveals microsecond nanoscopic protein motion on a live cell membrane. Nat. Photonics.

[130] Taylor R.W., Sandoghdar V. (2019). Interferometric Scattering Microscopy: Seeing Single Nanoparticles and Molecules via Rayleigh Scattering. Nano Lett..

[131] Young G., Kukura P. (2019). Interferometric Scattering Microscopy. Annu. Rev. Phys. Chem..

[132] Li N., Canady T.D., Huang Q., Wang X., Fried G.A., Cunningham B.T. (2021). Photonic resonator interferometric scattering microscopy. Nat. Commun..

[133] Merryweather A.J., Schnedermann C., Jacquet Q., Grey C.P., Rao A. (2021). Operando optical tracking of single-particle ion dynamics in batteries. Nature.

[134] Heermann T., Steiert F., Ramm B., Hundt N., Schwille P. (2021). Mass-sensitive particle tracking to elucidate the membrane-associated MinDE reaction cycle. Nat. Methods.

[135] Kashkanova A.D., Blessing M., Gemeinhardt A., Soulat D., Sandoghdar V. (2022). Precision size and refractive index analysis of weakly scattering nanoparticles in polydispersions. Nat. Methods.

[136] Küppers M., Albrecht D., Kashkanova A.D., Lühr J., Sandoghdar V. (2023). Confocal interferometric scattering microscopy reveals 3D nanoscopic structure and dynamics in live cells. Nat. Commun..

[137] Dahmardeh M., Mirzaalian Dastjerdi H., Mazal H., Köstler H., Sandoghdar V. (2023). Self-supervised machine learning pushes the sensitivity limit in label-free detection of single proteins below 10 kDa. Nat. Methods.

